# The use of non-speech oral-motor exercises among Indian speech-language pathologists to treat speech disorders: An online survey

**DOI:** 10.4102/sajcd.v62i1.82

**Published:** 2015-02-10

**Authors:** Roha M. Thomas, Ramesh Kaipa

**Affiliations:** 1 Department of Communication Sciences and Disorders, Oklahoma State University, United States of America

## Abstract

**Objective:**

Previous surveys in the United States of America (USA), the United Kingdom (UK), and Canada have indicated that most of the speech-language pathologists (SLPs) tend to use non-speech oral-motor exercises (NSOMEs) on a regular basis to treat speech disorders. At present, there is considerable debate regarding the clinical effectiveness of NSOMEs. The current study aimed to investigate the pattern and extent of usage of NSOMEs among Indian SLPs.

**Method:**

An online survey intended to elicit information regarding the use of NSOMEs was sent to 505 members of the Indian Speech and Hearing Association. The questionnaire consisted of three sections. The first section solicited demographic information, the second and third sections solicited information from participants who did and did not prefer to use NSOMEs, respectively. Descriptive statistics were employed to analyse the responses that were clinically relevant.

**Results:**

A total of 127 participants responded to the survey. Ninety-one percent of the participants who responded to the survey indicated that they used NSOMEs.

**Conclusion:**

The results suggested that the percentage of SLPs preferring to use NSOMEs is similar to the findings of surveys conducted in the USA, the UK, and Canada. The Indian SLPs continue to use NSOMEs based on a multitude of beliefs. It is important for SLPs to incorporate the principles of evidence-based practice while using NSOMEs to provide high quality clinical care.

## Introduction

Non-speech oral-motor exercises (NSOMEs) refer to oral activities that are believed to influence speech production without actually executing speech (Forrest, 2002). They include activities like lateral tongue sweeps, pursing and puckering of lips, puffing of cheeks, blowing, and sucking. Speech-language pathologists (SLPs) in many countries continue to use NSOMEs to treat various speech disorders (Hodge, Salonka & Koollias, 2005; Lof & Watson, 2008; Mackenzie, Muir & Allen, 2010). Clark (2003) indicated that most of the SLPs tend to use NSOMEs to treat speech deficits associated with Motor Speech Disorders (MSDs). The term MSDs includes the dysarthrias and apraxia of speech (AOS). Dysarthria refers to a group of neurologic speech disorders that reflect abnormalities in the strength, speed, range, steadiness, tone or accuracy of movements required for the breathing, phonatory, resonatory, articulatory, or prosodic aspects of speech production (Duffy, 2013). On the other hand, AOS refers to a motor planning and programming disorder in which the volitional production of speech is compromised (Theron, Van der Merwe, Robin & Groenewald, 2009).

The use of NSOMEs in the treatment of speech disorders has been one of the most debated topics within the scope of communication disorders (McCauley, Strand, Lof, Schooling & Frymark, 2009). Research carried out in the past two decades has searched for experimental evidence regarding the clinical utility of NSOMEs, and has resulted in equivocal results (McCauley, Strand, Lof, Schooling & Frymark, 2009).

A recurring theme in studies investigating the clinical utility of NSOMEs is the possible transfer and/or generalisation of treatment effects of NSOMES to speech production (Hodge, 2002; Weismer & Liss, 1991). The above authors opined that treatment of speech disorders should be task specific (i.e. using speech activities to treat speech disorders), rather than using NSOMEs to treat speech deficits. On the other hand, Clark (2003) emphasised the potential applications of NSOMEs in treatment of specific speech disorders. It is essential for clinicians to understand the theoretical underpinnings of specific NSOMEs (e.g., blowing, sucking, lip puckering), and the type of speech disorder they could be applied to. For example, strengthening exercises could be beneficial in treatment of an execution disorder like dysarthria, rather than treating AOS, which is a speech motor planning disorder. In spite of this ongoing debate surrounding the use of NSOMEs, a majority of the SLPs around the world continue to use NSOMEs based on anecdotal evidence.

Lof and Watson (2008) surveyed 2000 SLPs in the USA, of which 537 SLPs responded, a response rate of 27.5%. The results indicated that 85% of SLPs used NSOMEs to treat speech disorders. Mackenzie, Muir, and Allen (2010) surveyed the use of NSOMEs in the treatment of acquired dysarthria among SLPs in Scotland, Wales, and Northern Ireland. Postal questionnaires were sent out to 341 SLPs, and yielded responses from 191 SLPs, a response rate of 56%. The results of the survey indicated that 81% of the SLPs used NSOMEs in the treatment of acquired dysarthria. A similar study in Canada revealed that 85% of SLPs preferred to use NSOMEs to treat speech disorders (Hodge, Salonka & Koollias, 2005). Recently, McCauley, Strand, Lof, Schooling, and Frymark (2009) performed a systematic review of the evidence for using NSOMEs to improve speech production. The results of the review indicated that there is insufficient evidence to refute or support the use of NSOMEs to improve speech production.

Over the past few years, there has been an emphasis that the application of NSOMEs should be guided by evidence-based practice (Clark, 2003; Lass & Pannbacker, 2008; Muttiah, Georges & Brackenbury, 2010). The term ‘evidence-based practice’ (EBP) refers to using the best, research-proven assessment and treatment techniques to deliver the most effective services to patients (American Speech, Language, and Hearing Association [ASHA], 2005). According to the ASHA's position statement on EBP:

the goal of EBP is the integration of: (a) clinical expertise, (b) best current evidence, and (c) client values to provide high-quality services reflecting the interests, values, needs, and choices of the individuals we serve.

Even though, EBP emphasises the integration of all the above three aspects, it is possible that clinicians and academic researchers could differ on these three aspects. The clinicians, on the one hand, could be focused just on the clinical expertise and their personal opinions. Academic researchers, on the other hand, could be focused on finding the current evidence, neglecting the clinical perspective of the EBP. Hence, both researchers and clinicians have an obligation to work together to deliver the best practice for all the patients encountered.

At present, there is insufficient evidence to support or refute the use of NSOMEs. Until well-grounded research is conducted to prove or disprove the use of NSOMEs, it is essential to consider the clinical perspective of SLPs with regard to the use of NSOMEs, which is one of the three aspects of EBP. Even though SLPs continue to use NSOMEs on a regular basis, it is important for them to understand the scientific rationale behind the application of NSOMEs, and the speech disorders that can potentially benefit from NSOMEs. Currently, there is limited information as to why SLPs prefer to continue using NSOMEs. The scanty data available is based on the surveys conducted in the USA, Canada, and the UK. There are no data regarding the use of NSOMEs among SLPs in India. In India, there are approximately 1500 SLPs (Indian Speech & Hearing Association [ISHA], 2011) serving a population of approximately 1.2 billion (Office of the Registrar General & Census Commissioner, India, 2011). The decisions regarding the use of NSOMEs among Indian SLPs can have a major impact on clinical practice in India. Hence, the rationale of the current study was to investigate the extent of usage of NSOMEs among Indian SLPs, and if the application of NSOMEs is guided by scientific evidence. Specifically, the current study aimed to answer the following four research questions, (1) what is the percentage of SLPs using NSOMEs to treat speech disorders in India, and rationale for the use of NSOMEs, (2) what are the types of speech disorders for which NSOMEs are used, (3) what are the different types of NSOMEs used to treat speech disorders, and (4) what is the percentage of SLPs not preferring to use NSOMEs, and the rationale for not using NSOMEs.

## Methods

The general purpose of the current study was to investigate the views and usage of NSOMEs among Indian SLPs. Specifically, the current study aimed to explore the demographics regarding the percentage of SLPs using, and not using NSOMEs, the type of NSOMEs used to treat speech disorders, and the type of speech disorders for which NSOMEs were used.

### Research design

The current study implemented a cross-sectional survey research design to achieve the above aims. A cross-sectional design provides a snapshot of a set of characteristics that exist in a population at a specific point of time (Levin, 2006). Survey research involves collecting information regarding a specific topic from a group of individuals through their responses to the questions. Survey designs are relatively inexpensive, and can potentially elicit responses from individuals in remote locations (Bartlett, Kotrlik & Higgins, 2001). For the above reasons, the researchers deemed the cross-sectional survey design to be appropriate for this study.

#### Development of the online survey

A questionnaire was created using the Qualtrics^©^ software (Qualtrics, Provo, UT, 2005) for the purpose of online data collection. The questions listed on the survey were prepared specifically to suit the nature of academic training related to communication disorders in India, and the work environment of Indian SLPs. The questionnaire comprised three sections of 27 questions in total, and took approximately 10 min to complete. The questionnaire included multiple-choice and yes/no questions. A majority of the questions allowed the respondents to choose multiple answers from the provided options. However, a small number of questions required the participants to choose just one option. The first section of questions solicited demographic details of the participants such as the type of work setting, work experience, education, gender, and area of clinical specialty. The second section consisted of questions related to the use of NSOMEs by the participants including, though not limited to clientele information, speech disorders for which NSOMEs were used, type of NSOMEs used, and frequency of use of NSOMEs. If the participants indicated that they did not use NSOMEs, they were directed to the third section of questions. The third section elicited information from respondents who did not use NSOMEs in their clinical practice (please see Appendix for the complete questionnaire).

#### Procedure

An e-mail was sent to 505 members listed in the online directory of the ISHA. The e-mail consisted of an introductory message that invited the respondents to participate in the survey, the web link to the online survey questionnaire, and instructions to successfully complete the survey. In addition, the details of the survey and the link to the survey were posted on the research webpage of the ISHA.

#### Participants

A total of 127 individuals responded to the online survey. All the participants provided consent to participate in the current study. With regard to the education level of the participants, 75% had completed a master's degree in Communication Sciences and Disorders, 16% had completed a Bachelor's degree, a small number of participants had obtained a Ph.D. (6%), and 4% obtained a Master's degree from a different field (e.g., Psychology). A majority of the participants (74%) had 0–5 years of clinical experience. A small percentage (18%) reported that they had clinical experience ranging from 6–10 years, whilst a handful of participants (2%) reported that they had clinical experience ranging from 16–20 years, and another 2% had experience of more than 20 years. With regard to work settings, a little less than half the number of participants (48%) worked in Universities, another 48% of the participants worked in hospitals, around 34% worked in private practice, and only 7% worked in schools. Some of the respondents indicated that they worked in multiple settings, leading to an overlap of the responses. The male participants accounted for 31% of the respondents. The demographic information related to participants is presented in Table 1.

**Table 1.** Demographic details of the participants.

Demographic details of the participants	Response (%)
**Sex of the participants**	
Male	31
Female	69
**Highest level of education**		
Bachelor's	16
Master's	75
Doctoral (PhD)	6
Other	4
**Length of the clinical experience**	
0–5 years	74
6–10 years	18
11–15 years	4
16–20 years	2
**Nature of the work setting**	
School	7
Hospital	48
College/University	48
Private Clinic	34
Other	4
**Type of Master's program**	
M.Sc. (Audiology)	2
M.Sc. (Speech-Language Pathology)	14
M.Sc. (Speech & Hearing)	18
MASLP (Dual)	60
Other	7
**Type of speech disorders on the case load**	
Speech sound disorders	72
Motor speech disorders	74
Voice disorders	71
Fluency disorders	75
Developmental language disorders	81
Adult language disorders	67
Resonance disorders	36
Swallowing/feeding disorders	52
Others	3

#### Ethical clearance

The Institutional Review Board at the authors’ institution approved this study. Informed consent was obtained from all the participants. The participants’ responses were completely anonymous to protect their identity. The participants were made aware that they could withdraw their participation at any stage of the survey.

#### Data analysis

Previous survey questionnaires that were used to collect data about the use of NSOMEs were compared to the current study's questionnaire to ensure criterion validity. An Indian SLP with five years of clinical experience reviewed the content of the questionnaire in order to ensure that it was appropriate for eliciting valid information regarding the use of NSOMEs. This served as a measure of content validity. Unfortunately, the test-retest reliability of this survey could not be determined as the same respondents were unable to take the survey for the second time.

Descriptive statistics were used to analyse the obtained data. The participants’ responses to each item on the questionnaire were aggregated and converted to a percentage by the Qualtrics^©^ software. In addition, the strength of association between certain variables on the questionnaire was measured using the Chi-square test of association. The alpha level was set at 0.05.

## Results

A total of 127 participants responded to the online survey, yielding a response-rate of 25.4%. The aggregated responses of participants are reported under two main sections: participants preferring to use NSOMEs, and participants not preferring to use NSOMEs.

### Participants preferring to use NSOMEs

With a view to gaining a better insight regarding the clinicians’ perspective and rationale for using NSOMEs, the results of the following aspects that are relevant to the aim of this study are reported: current or past use of NSOMEs in speech therapy, length of usage of NSOMEs, conditions for which NSOMEs are used, different disorders for which NSOMEs are used, types of NSOMEs used, types of materials used for NSOMEs, frequency of usage of NSOMEs, reasons for believing that NSOMEs are effective, and future use of NSOMEs. The percentage responses that are reported below are based on the responses from participants who preferred to use NSOMEs.

#### Current or past usage of NSOMEs

A majority of the participants (91%) indicated that they either used, or had been using NSOMEs as a speech therapy technique.

#### Length of using NSOMEs

With regard to the length of using NSOMEs as a speech therapy technique, a small number of participants (8%) reported using NSOMEs for less than one year. Around 13% indicated they were using NSOMEs for 3–4 years. There was a roughly equal representation of participants who had been using NSOMEs for 1–2 years (20%), 2–3 years (19%), 4–5 years (22%), and more than 5 years (17%).

#### Conditions for which NSOMEs were used

Most of the participants (96%) reported that they were using NSOMEs to improve the motor aspect of the articulators (e.g., to improve the strength, tonicity). Around 65% used NSOMEs to improve the sensory deficits of oral structures (e.g., hypersensitivity). Some of the participants (61%) also indicated that they used NSOMEs to manage feeding problems in children. A sizable number of participants (83%) also reported that they used NSOMEs with their clients to control drooling. A small number of the participants (8%) indicated that they used NSOMEs for other conditions such as speech sound disorders, developmental AOS and child language disorders such as autism (to improve oral sensory issues).

#### Types of speech disorders for which NSOMEs were used

An overwhelming number of participants (92%) used NSOMEs to treat MSDs. This was followed by 82% of the participants using NSOMEs to treat swallowing disorders. Approximately half the number of participants (51%) used NSOMES to treat speech sound disorders. Around 27%, 29%, and 21% of the participants used NSOMEs to treat clients with developmental language disorders, adult language disorders, and resonance disorders, respectively. In addition, a small percentage of the participants (7%) used NSOMEs to treat voice disorders and another 7% of the participants used NSOMEs to treat fluency disorders.

#### Types of NSOMEs used

Most of the NSOMEs were used by an equal number of participants. Lip puckering was used by a majority of the participants (90%), followed by blowing (87%) and puffing of cheeks (87%). Lateral lip movements, lateral tongues sweeps, alternative lip puckering and/or smiling, sucking and vertical tongue movements were used by 73%, 80%, 83%, 75%, and 81% of the participants, respectively.

#### Types of materials used for NSOMEs

The most frequently used material for NSOMEs was straw, which was used by 84% of the participants. This was followed by paper strips (83%) and brushes (83%). Blowing whistles, balloons and cotton balls were used by 76%, 67%, and 56% of the participants, respectively. Horns were the least frequently used materials (25% of the participants).

#### Frequency of usage of NSOMEs

A little more than half the number of participants (56%) indicated that they used NSOMEs occasionally (25–50% of the therapy sessions). Around 40% of the participants indicated that they used NSOMEs frequently (more than 75% of the therapy sessions). Only a small number of participants (5%) indicated that they used NSOMEs rarely (less than 10% of the sessions).

#### Reasons for believing that NSOMEs are effective

Most of the participants (84%) believed that NSOMEs seemed to be effective, because they helped to strengthen the articulators, thereby improving speech intelligibility. A little less than half the number of participants (43%) indicated that they used NSOMEs as they thought they are useful for improving the sensory problems of the oral-facial region. Around 34% of the participants reported that they used NSOMEs, as they have read journal articles or book chapters about the efficacy of NSOMEs. Approximately 31% of the participants believed that speech develops from non-speech tasks (like blowing), and hence using NSOMEs to treat speech disorders seemed to be logical. Finally, 31% of the participants believed that NSOMEs were effective based on their personal experience.

#### Use of NSOMEs in future

Around two-thirds of the participants (75%) reported that they would continue to use NSOMEs in future along with other speech therapy techniques. A small number of participants (13%) mentioned that they would continue to use NSOMEs for a long time. Another 13% of the participants indicated that they might use NSOMEs for some time in future and would discontinue them if there are better speech therapy techniques. The responses of the participants who preferred to use NSOMEs as a speech therapy technique are presented in Table 2.

**Table 2.** Findings related to participants preferring to use NSOMEs.

Findings	Response (%)
**Use of NSOMEs in speech therapy**	
Yes	91
No	9
**Knowledge about NSOMEs**	
Undergraduate/Postgraduate classes	84
Colleagues/Seniors	37
Continuing education events	15
Text books/Research articles	58
**Length of using NSOMEs as a speech therapy technique**	
0–1 years	8
1–2 years	20
2–3 years	19
3–4 years	13
4–5 years	22
More than 5 years	17
**Type of disorders treated using NSOMEs**	
Speech sound disorders	51
Motor speech disorders	92
Resonance disorders	21
Voice disorders	7
Fluency disorders	7
Developmental language disorders	27
Adult language disorders	29
Swallowing/feeding disorders	82
**Type of NSOMEs used**	
Lip puckering	90
Lateral Lip movements	73
Alternative lip puckering/smiling	83
Smiling/ exaggerated smiling	71
Lateral tongue sweeps	80
Vertical tongue movements	81
Blowing	87
Sucking	75
Puffing of cheeks	87
Others	17
**Type of materials used for NSOMEs**	
Straws	84
Cotton balls	56
Paper strips	83
Balloons	67
Horns	25
Blowing whistles	76
Brushes	83
**Conditions for which NSOMEs were used**	
To improve motor aspect of articulators	96
Treating oral-sensory issues	65
Treating feeding problems	61
To control drooling	83
Other conditions	8
**Frequency of use of NSOMEs**	
Frequently (>75% of the sessions)	40
Occasionally (25-50% of the sessions	56
Rarely (< 10% of the sessions)	5
**Use of NSOMEs in future**	
Continue to use for a long time	13
Use along with other speech therapy techniques	75
Use for a while and discontinue if better treatment techniques are available	13
Not planning to use NSOMEs in future	0
**Reasons for believing that NSOMEs are effective**	
Speech develops from non-speech tasks, so NSOMEs improves speech	31
NSOMES helps in developing muscle strength, thereby improving speech intelligibility	84
Read research articles/textbooks about the efficacy of NSOMEs	34
Personal experiences	31
Improves sensory problems of the oral facial region	43

### Participants not preferring to use NSOMEs

With regard to the participants not preferring to use NSOMEs, the results of the following aspects that are relevant to the aim of the current study are reported: current and/or past use of NSOMEs, rationale for not using NSOMEs, awareness of research discouraging the use of NSOMEs, factors that would encourage consideration of NSOMEs. The percentage responses that are reported below are based on the responses from participants who did not prefer to use NSOMEs.

#### Current and/or past usage of NSOMEs

Around 9% of the participants who responded to the survey reported that they did not use NSOMEs as a speech therapy technique.

#### Rationale for not using NSOMEs

Among the 9% of the participants who did not prefer to use NSOMEs, the main reasons cited for not using NSOMEs were: (1) around half the number of participants (50%) were not seeing clients who required the use of NSOMEs, (2) personal experience (38%), (3) lack of research supporting the use of NSOMEs (38%), and (4) attending continuing education events that did not encourage the use of NSOMEs (25%).

#### Awareness of research discouraging the use of NSOMEs

More than half the number of participants (63%) indicated that they were aware of research that discouraged the use of NSOMEs as a speech therapy technique.

#### Factors that would encourage consideration of NSOMEs

All the participants (100%) reported that they would consider using NSOMEs if there was adequate evidence supporting the use of NSOMEs in clinical practice. Around two-thirds of the participants (75%) indicated that they would consider NSOMEs if they had personal experience of having success in using NSOMEs with their clients. A small number of participants (25%) mentioned that they would consider using NSOMEs in future if they witnessed an increasing number of clinicians using NSOMEs.

The responses of the participants who did not prefer to use NSOMEs are presented in Table 3.

**Table 3.** Findings related to participants not preferring to use NSOMEs.

Findings	Response (%)
**Reason for not using NSOMEs in speech therapy**	
Not convinced based on personal experience	38
Not useful	25
No literature that supports the use	38
Not beneficial as learned from colleagues/lectures	0
Others	50
**Awareness about the research that discourages the use of NSOMEs**	
Yes	63
No	38
**Factors influencing the use of NSOMEs in future**	
Practice based evidence of NSOMEs	100
Personal success of using NSOMEs	75
An increase in number of SLPs using NSOMEs	25
I will not consider using NSOMEs	0
Others	0

In addition to the participants’ responses to specific items on the questionnaire, the authors were interested to see if there was an association between the length of participants’ clinical experience and preference to use NSOMEs. The results of a Chi-square test revealed that there was a significant association between length of clinical experience and preference to use NSOMEs, χ^*2*^ (4, *N* = 99) = 11.01, p < 0.05.

## Discussion

The findings of past studies investigating the use of NSOMEs among SLPs in the USA, the UK, and Canada revealed that a significant number of SLPs preferred to use NSOMEs. The current study aimed to investigate the pattern and extent of usage of NSOMEs among Indian SLPs. Some of the findings of the current study align with previous studies of similar nature. The clinical findings relevant to the four primary research questions that were posed at the beginning of this paper are discussed below.

### What is the percentage of SLPs using NSOMEs to treat speech disorders, and rationale for using NSOMEs?

Around 91% of the participants indicated that they used NSOMEs to treat speech disorders. Similar estimates have been reported in the USA (85%) (Lof & Watson, 2008), the UK (81%) (Mackenzie, Muir, and Allen, 2010), and Canada (85%) (Hodge, Salonka & Koollias, 2005). There are several factors that can be attributed to the high percentage of SLPs using NSOMEs in India. Firstly, NSOMEs are relatively easy to be administered with clients. It is typical for clinicians in India working in private clinics and hospitals to have a large caseload. The availability of oral-motor kits makes it convenient for clinicians to administer NSOMEs during therapy sessions rather than incorporating activities that require extensive preparation. Secondly, the clinical practice of speech-language pathology in India has been influenced by Western countries to some extent in the form of webinars, textbooks, and research articles. This can be another reason for the high percentage of Indian SLPs using NSOMEs similar to SLPs in Western countries. Finally, the idea of NSOMEs is more of a cultural transmission from one generation of SLPs to another. This cultural transmission is a likely reason why NSOMEs have survived in India for so many years.

With regard to the rationale for using NSOMEs, an overwhelming majority of the participants indicated that they used NSOMEs as they believed that they helped in developing the muscle strength of the articulators. Even though the use of NSOMEs as a speech therapy technique has been fraught with controversy, it is likely that NSOMEs could be useful in a small percentage of clinical population who present with articulatory weakness. (Clark, 2003; Duffy, 2013). A common argument against using NSOMEs to strengthen the articulators is that individuals require only a small proportion of their oral muscular force to produce speech (Muller, Milenkovic & MacLeod, 1985). According to this notion, an individual with reduced muscular force of the tongue will still be able to produce the necessary force required for normal speech production. However, Luschei (1991) argued against this notion. He stressed that, whilst high force may not be required during lingual speech movements, adequate power might be a prerequisite for agile articulatory movements. In this case, strength training to improve the power rather than the force could prove beneficial to a patient with lingual weakness (Clark, 2003). Duffy (2013) also recommended using NSOMEs only to treat articulatory deficits that are associated with weakness. Thus, the rationale expressed by a majority of the Indian SLPs for using NSOMEs seems to be in line with previous literature that advocated the use of NSOMEs to treat speech deficits associated with oral weakness.

Less than half the number of participants indicated that they used NSOMEs because they thought they improved the sensory problems of the oral-facial region. Marshalla (1985) proposed that the sensorimotor experience in early childhood serves as a foundation for speech development. Therefore, it is logical to assume that sensory deficits contribute to deficits in speech production. However, remediating oral-sensory deficits to improve the motoric aspect of speech production seems to be questionable, and needs to be further explored. Previous studies have not been able to establish a relationship between sensory acuity and speech proficiency. The clinicians need to be aware that treating oral-sensory issues in order to improve the speech production could be a futile attempt.

Some of the other reasons provided by the participants for using NSOMEs were: speech develops from non-speech tasks (like blowing), reading research articles or book chapters about the efficacy of NSOMEs, and personal experience. Previous research has revealed that during the early stages of development, the movements for a motor pattern for non-speech activities such as chewing tend to be different from motor pattern during speech (Moore, Smith & Ringel, 1988). The rationale expressed by some of the SLPs that speech develops from non-speech activities does not seem to hold ground based on the existing evidence. In general, the current study reveals that SLPs in India continue to use NSOMEs based on a multitude of beliefs. At present, the only rationale that seems to be supported by some form of evidence is that NSOMEs could be helpful in strengthening of the articulators. However, SLPs should be aware that this could be applicable only to disorders like flaccid dysarthria that are associated with weakness of the articulators.

### What are the types of speech disorders for which NSOMEs are used?

The survey results indicated that NSOMEs were used more frequently by Indian SLPs to treat MSDs. The questionnaire in the current study did not differentiate the specific types of MSDs for which the NSOMEs were used (e.g., dysarthria vs. AOS). However, the questionnaire had a separate option titled ‘other’, where the participants were able to indicate the specific disorders and/or conditions for which they used NSOMEs. A small percentage of the participants did indicate that they used NSOMEs to treat AOS.

Weakness is one of the common motor impairments that accompany both developmental (e.g., cerebral palsy), as well as acquired dysarthria (e.g., following a stroke) (Clark, Henson, Barber, Stierwalt & Sherrill, 2003; Dworkin & Aronson, 1986; Dworkin, Aronson & Mulder, 1980; Dworkin & Hartman, 1979; Gentil, Perrin, Tournier & Pollak, 1999; Langmore & Lehman, 1994; Murdoch, Attard, Ozanne & Stokes, 1995). It is not surprising that NSOMEs intended for strength training are frequently applied to treat an execution disorder like dysarthria. In fact, the findings of Lof and Watson (2008) revealed that SLPs in the USA used NSOMEs more frequently to treat dysarthria than other disorders. However, applying NSOMEs to treat a planning-based disorder like AOS may be questionable, as there is no consensus between the control of non-speech oral movement and planning speech movements (Ziegler, 2003). It is easy for clinicians to be misguided by several commercially available resources that recommend NSOMEs as an intervention for individuals with AOS. However, clinicians should be critical in generalising the benefits of NSOMEs to other types of MSDs such as AOS.

Participants also indicated that they used NSOMEs to treat speech sound disorders, swallowing and/or feeding disorders, developmental language disorders, resonance disorders, voice, as well fluency disorders. At first glance, these findings may seem to be surprising. However, it is possible that the participants reported using NSOMEs to treat oral motor deficits that co-occurred with the above disorders rather than the primary disorder, *per se*. It is not uncommon to find literature that reports oral motor deficits associated with speech sound disorders (Chapman-Bahr, 2001), swallowing disorders (Adams, Callister, & Mathisen, 2011) and autism spectrum disorders (ASDs) (Belmonte *et al*. 2013). For example, Belmonte *et al*. found a correlation between oral motor deficits and the speech and language abilities in a cohort of children with ASDs. A caveat on using NSOMEs to treat different types of speech disorders is that the outcomes of NSOMEs could not only vary when applied to different speech disorders, but could prove to be harmful as well. The decision to apply NSOMEs to a certain speech disorder should begin with analysing the pathophysiology of the speech disorder. If the underlying pathophysiology does not tend to be at the level of motor speech execution, the potential attempt at using NSOMEs should be avoided.

### What are the types of NSOMEs used to treat speech disorders?

It was found that lip puckering was used by a majority of the participants (90%). Other NSOMEs such as lateral lip movements, lateral tongue sweeps, vertical tongue movements, blowing, sucking, puffing of cheeks, and smiling were used on an almost equal basis. The lack of empirical support for the use of NSOMEs makes it necessary for SLPs to understand the influence of different NSOMEs on the oral muscular physiology, and how they can act on the underlying speech impairments.

Before SLPs can decide on using a specific oral-motor exercise, they should evaluate the physiological impact of that exercise on the underlying speech impairment. For example, if the goal is to improve the lingual weakness, an appropriate strength training protocol (in the form of isometric and/or isotonic exercises) should be chosen. The ultimate goal of a strength-training programme is to enhance strength, endurance, and power of the affected muscles. However, this enhancement is only possible when the muscle is taxed beyond its normal workload (Clark, 2003). In the present study, the participants were not provided with an opportunity to express as to why they preferred using a certain exercise over other exercises; this is a limitation that needs to be addressed in future research. In general, SLPs need to be rational and choose NSOMEs that could have a positive impact on the underlying impairment rather than choosing NSOMEs that tend to be more popular with other SLPs.

### What is the percentage of SLPs preferring not to use NSOMEs, and their justification for this choice?

A small percentage of participants (9%) indicated that they did not prefer to use NSOMEs. Around half the number of participants stated that they did not use NSOMEs as they were seeing clients who did not require the application of NSOMEs. The other reasons provided by participants for not using NSOMEs were lack of evidence supporting the use of NSOMEs, and personal experience. However, a majority of these individuals considered using NSOMEs in the light of adequate evidence supporting their use. The rationale expressed by the participants for not using NSOMEs seems to be in agreement with the published literature criticising the use of NSOMEs (Forrest, 2002; Loff & Watson, 2008). Over the past few years, an increasing number of researchers have expressed concern over the use of NSOMEs as a speech therapy technique (Lass & Pannbacker, 2008; Ruscello, 2008). Recent research has subjected NSOMEs to the test of ‘EBP’, and the results have indicated that there is minimal evidence for the success of NSOMEs as a speech therapy technique (Lass & Pannbacker, 2008; Ruscello, 2008; Powell, 2008). In addition, there has been a plethora of published literature criticising the theoretical foundations of NSOMEs. McCauley, Strand, Lof, Schooling, and Frymark (2009) recommended clinicians to use speech treatments of which the efficacy is already established, rather than using NSOMEs.

The usage of NSOMEs seems to be widespread owing to the clinical perspectives of SLPs, rather than based on the scientific evidence. It is not surprising that the participants in the current study were reluctant to use NSOMEs, as they were not convinced of the treatment efficacy of NSOMEs.

### Association between the length of clinical experience and preference to use NSOMEs

As data were collected from participants with clinical experience ranging from almost no experience to more than 20 years, the authors were interested in exploring whether clinical experience had any influence on decision-making regarding the use of NSOMEs. The results indicated that there was significant association between the length of clinical experience and the preference to use NSOMEs. A majority of the participants who preferred to use NSOMEs had clinical experience ranging from 0–5 years. The participants’ preference to use NSOMEs decreased as their clinical experience increased.

Two reasons can be accounted for this finding. Firstly, it is possible that participants who had no or limited clinical experience (0–5 years) experimented with the outcomes of NSOMEs by using them as a speech therapy technique on a regular basis. Over the course of years, the participants could have realised there were limited benefits in using NSOMEs to treat speech disorders. This could be one of the reasons why participants with more clinical experience did not prefer to use NSOMEs. Secondly, most of the SLPs (74%) who participated in the study had clinical experience ranging from 0–5 years. The data could have been skewed by including very few participants with more than five years years of clinical experience, which would have made it appear as if participants with limited clinical experience use NSOMEs regularly compared to participants with more clinical experience. Hence, this finding should be extrapolated with caution.

## Limitations

There were some obvious limitations in the current study that could have had a bearing on the results. The first limitation is a low response rate from the prospective participants. The responses of the participants discussed in this study do not represent the views of the entire SLP community in India. Hence, the information regarding the clinical application of NSOMEs among other SLPs remains unknown. The second limitation is that the questionnaire used in this study did not differentiate the application of NSOMEs between acquired and congenital disorders, as well as between dysarthria and AOS. This could have provided more information regarding how participants differ in the application of NSOMEs to treat a congenital vs. acquired speech disorder, and whether SLPs understand the rationale in applying NSOMEs to an execution based disorder (dysarthria) versus motor planning disorder (AOS). The third limitation is the inclusion of a limited number of participants with varied clinical experience. A majority of the participants in the current study had limited clinical experience. Recruiting participants with different lengths of clinical experience would have allowed the authors to investigate whether the attitude towards the use of NSOMEs changes with increasing clinical experience.

## Conclusion

The controversy surrounding the use of NSOMEs has been an ongoing issue for the past several years, and will continue until both sides of the argument agree to work together with the intention of answering unsolved questions. The application of EBP is essential to the growth of a clinical profession like speech-language pathology. EBP encourages the integration of clinical expertise, best available research evidence, and the client's values (Muttiah, Georges, & Brackenbury, 2011). In the process of applying EBP to the use of NSOMEs, two distinct groups have emerged: clinicians and researchers.

Muttiah, Georges, and Brackenbury (2011) mentioned that there is considerable difference of opinion between clinicians and researchers regarding the implementation of NSOMEs. Clinicians, on the one hand, tend to be concerned with their clinical perspective, and could neglect searching for quality evidence. On the other hand, researchers tend to spend a lot of time in laboratories, and have minimal contact with actual practitioners. Both researchers as well as clinicians have to realise that they have a shared responsibility in promoting EBP with regard to the use of NSOMEs. Even though a majority of the SLPs continue to use NSOMEs, it is essential for them to document the outcomes and share them with researchers in order to increase transparency in clinical practice. The findings of the current study revealed that a majority of SLPs are inclined to use NSOMEs in spite of the surrounding controversy. An ideal way to resolve this controversy would be to conduct well-designed single case experimental studies that evaluate the treatment benefits of NSOMEs.
